# ^1^H, ^13^C and ^15^N chemical shift assignments of Rubella virus macro domain in the free and in the ADPr bound state

**DOI:** 10.1007/s12104-025-10227-4

**Published:** 2025-04-05

**Authors:** Danai Moschidi, Nikolaos K. Fourkiotis, Christos Sideras-Bisdekis, Aikaterini C. Tsika, Georgios A. Spyroulias

**Affiliations:** Department of Pharmacy, University of Patras, Patras, 26504 Greece

**Keywords:** Rubella virus (RuV), Macro domain (MD), ADP-ribosylation, Solution NMR spectroscopy

## Abstract

Prokaryotes, eukaryotes, and certain viruses with positive single-stranded RNA genomes are among the forms of life that have been found to possess macro domains (MDs). There are claims that viral MDs inhibit the immune response mediated by PARPs, such as PARP12 and PARP14, and are involved in the formation of the viral replication transcription complex (RTC). Rubella virus (RuV) is included in this group of viruses. Its MD acts as an “eraser” of the posttranslation modification (PTM) ADP-ribosylation by binding to and hydrolyzing ADP-ribose (ADPr) from ADP-ribosylated substrates including proteins and nucleic acids. Consequently, it represents an attractive pharmacological target. Currently, no inhibitors exist for RuV MD’s de-ADP-ribosylation activity, which may play a crucial role in viral replication and pathogenesis, as observed in severe acute respiratory syndrome coronavirus (SARS-CoV) and Chikungunya virus (CHIKV). RuV remains a serious threat, particularly to unvaccinated children, with approximately 10,000 of the 18,000 global cases in 2022 reported in Africa. Alarmingly, no FDA-approved drugs are available for RuV treatment. In this study, we present the almost complete NMR backbone and side-chain resonance assignment of RuV MD in both free and ADPr bound forms, along with the NMR chemical shift-based secondary structure element prediction. These findings will support the efficient screening of fragments or chemical libraries using NMR spectroscopy to identify compounds that are strong binders and potentially exhibit antiviral activity.

## Biological context

**Fig. 1:**
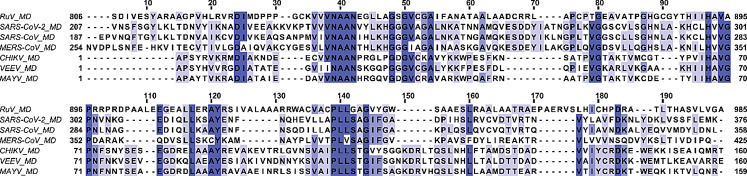
Sequence alignment of MDs of three viral families, *Matonaviridae* (RuV), *Coronaviridae* (SARS-CoV-2, SARS-CoV and MERS-CoV) and *Togaviridae* (CHIKV, VEEV and MAYV). Amino acid numbering is according to the native sequence of the multi-domain p150 for RuV MD and non-structural protein 3 (nsP3) for the rest. White color indicates non-conserved residues, light blue less conserved residues and dark blue conserved residues through all MDs.

Rubella virus (RuV) belongs to the *Matonaviridae* family and the genus *Rubivirus*, which includes two other viruses that infect only animals, the Ruhugu virus (RuhV) found in bats and the Rustrela virus (RusV) in rodents (Bennett et al. 2020; Mankertz et al. 2022). The symptoms of Rubella, a highly contagious virus that spreads through droplets, include fever, and coughing, while sometimes the patients also develop arthritis, painful joints, and a rash (Kilich et al. 2024). Even though there is a safe and cost-effective vaccine which protects against Rubella, there were reported approximately 18,000 cases of Rubella worldwide in 2022, according to the World Health Organization (WHO) ([WHO], 2024). Rubella is regarded as one of the world’s most common causes of death for children. It can also infect pregnant women during the first trimester, potentially causing fetal death or resulting in babies with congenital rubella syndrome (CRS) ([WHO], 2024). *Matonaviridae* family consists of enveloped viruses that possess a single-stranded positive-sense RNA genome ((+)ssRNA) of up to 10 kb in length and high in GC content, with RuV having (+)ssRNA close to 9.6 kb and 69%, respectively. RuV genome encodes three structural proteins from its 3’ORF, the capsid (C) and the two glycoproteins E1 and E2, and one non-structural protein (nsP) named p200 from its 5’ORF. p200 is further processed into two nsPs, the p90 and the p150 (Matthews et al. 2010), that are vital for the formation of replication-transcription complex (Marr et al. 1994). p90 consists of two protein domains, the helicase (Hel) and the RNA-dependent RNA polymerase (RdRp), while p150 contains three independent domains, the methyltransferase (MTase), the macro domain (MD) and the papain-like protease (PLpro) (Prasad et al. 2013; Cheong et al. 2022). Other (+)ssRNA viruses belonging to viral families, such as *Togaviridae*, *Coronaviridae,* and *Hepeviridae*, also possess MDs (Makrynitsa et al. 2015; Melekis et al. 2015; Li et al. 2016; Lykouras et al. 2018; Grunewald et al. 2019; Palazzo et al. 2019; Tsika et al. 2019, 2022; Leung et al. 2022; Politi et al. 2023). The MDs exhibit an α/β/α three-layered sandwich structure (Tsika et al. 2019), comprised of several Rossmann β-α-β motifs. These secondary structure elements are assembled in a central mixed β-sheet, consisting of 6–7 β*-*strands flanked by 4–5 α-helices, forming on one side of the molecule a positively charged cavity that is responsible for binding ADPr, nucleic acids, and other NAD^+^ derivatives (Cantini et al. 2020; Fourkiotis et al. 2022; Tsika, et al. 2022). Moreover, the structure of MDs closely resembles the structure of proteins that bind NAD^+^ derivatives-nucleic acids, such as leucine aminopeptidases (Allen et al. 2003). Today, many MD structures for a variety of organisms, either in their free or bound to ADPr or other small molecules forms, have been solved (Malet et al. 2009; Forst et al. 2013; Cho et al. 2016; Tsika et al. 2019; Michalska et al. 2020). This is also the case for Rubella virus, as the structures of RuV MD, both in free and ADPr bound states, have been recently solved through X-ray crystallography (Stoll et al. 2024). RuV MD structure resembles that of other viral MD domains in the MacroD-type family, such as the MD of Mayaro virus (MAYV) (Tsika et al. 2019) and SARS-CoV-2 (Alhammad et al., 2021), which belong to Alphaviruses and Coronaviruses, respectively (Stoll et al. 2024). Note that the RuV MD exhibits higher sequence identity with Alphaviruses MD than with Coronaviruses MD. It has the highest sequence identity to MAYV MD (32%) and the lowest to SARS-CoV MD (24%) (Fig. 1).

Regarding its activity, it was shown that RuV MD possesses the capacity to remove either single ADPr units (MAR) or ADPr chains (PAR), from substrates that have been ADP-ribosylated; these characteristics are also known as de-MARylation and de-PARylation, respectively, and the first is a feature of most of the viral MDs (Stoll et al. 2024). Viral MDs' ability to bind ADPr, its derivatives (e.g. PAR, OAADPr) and nucleic acids was initially not associated with any biological function until the de-MARylation activity of Hepatitis E virus (HEV) was discovered (Li et al. 2016). In recent years, their dual role as “readers” and “erasers” of ADP-ribosylation, in vitro and in vivo, has made them molecules of particular biological and medicinal interest (Fehr et al. 2016; Alhammad et al. 2023). Following infection by (+)ssRNA viruses, host cells enhance PARP-mediated ADP-ribosylation, whereas viral macro domains counteract this modification, potentially allowing the virus to evade antiviral defenses. As an example, PARP10 targets and MARylates the protease of CHIKV, rendering it inactive, while CHIKV reverses this PTM through its MD, by hydrolytically removing MAR (Krieg et al. 2023). This could be the case for more viral and/or host proteins in the context of antiviral response (Li et al. 2016; Alhammad and Fehr 2020; Fourkiotis et al. 2022). The fact that viral MDs capability of removing MAR from their substrates enhances their pathogenicity was supplemented by the discovery that their ability to simply bind free or bound to substrate ADPr affects the replication process of the viral genome (Abraham et al. 2020; Voth et al. 2021; Kerr et al. 2024). So, viral MDs like RuV MD are considered potential drug targets, and work is being performed to find new molecules that can inhibit their function (Berg et al. 2022).

The almost complete RuV MD backbone and side-chain resonance assignment is presented here in both free and ADPr bound forms. Even though the structures of RuV MDs in the free (PDB ID: 8P0C) and ADPr bound states (PDB ID: 8P0E) have previously been solved by X-ray crystallography, this NMR study provides important insights into the protein’s dynamics and allows the characterization of biomolecular interactions with potential ligands in a setting that is similar to its native in vivo environment.

## Methods and experiments

### Construct design

A codon-optimized gene (Uniprot accession number: Q99IE5) encoding the RuV MD (806–985 a.a., which is part of the protease/methyltransferase p150 polypeptide of the non-structural polyprotein p200) for expression in the heterologous system *E.coli* was obtained from GenScript (Piscataway, NJ). It was amplified with PCR and cloned into pET20b(+) expression vector between the restriction sites *Nde*I-*Xho*I. The polypeptide was expressed with cloning artifact amino acids, one at the N-terminal region (methionine) and two, leucine and glutamate, preceding the uncleavable His_6_-tag in the C-terminus. For the verification of the obtained construct DNA sequencing was used.

### Protein expression and uniform ^15^N and ^15^N/^13^C labeling

The plasmid expressing RuV MD was transformed into Rosetta™2(DE3) pLysS *E. coli* cells. A *Luria-Bertani* (LB) pre-culture that was inoculated with the above cells was grown overnight at 37 °C with shaking at 180 rpm. This pre-culture was then used to inoculate a 0.5 L M9 minimal medium (40 mM Na_2_HPO_4_, 22 mM KH_2_PO_4_, 8 mM NaCl) containing 0.5 g ^15^NH_4_Cl and 2 g unlabeled or ^13^C-D-glucose, 1 mL from a stock solution containing 0.5 mg/mL biotin and 0.5 mg/mL thiamine, 0.5 mL of 1 M Mg_2_SO_4_, 0.15 mL of 1 M CaCl_2_, 1 mL of trace elements solution (40 mM HCl, 50 mg/L FeCl_2_·4H_2_O, 184 mg/L CaCl_2_·2H_2_O, 64 mg/L H_3_BO_3_, 18 mg/L CoCl_2_·6H_2_O, 4 mg/L CuCl_2_·2H_2_O, 340 mg/L ZnCl_2_, 710 mg/L Na_2_MoO_4_·2H_2_O, 40 mg/L MnCl_2_·4H_2_O), 0.5 mL of 10X stock solution of BioExpress^®^ 1000 (U-^13^C, 98%; U-^15^N, 98%), 100 µg/mL ampicillin and 34 µg/mL chloramphenicol. The culture was incubated at 37 ˚C with shaking at 180 rpm until the OD_600_ was between 0.6 and 0.8 and adaptation at 18 ˚C followed for 2 h. IPTG to a final concentration of 1 mM was added to the culture, which finally was incubated overnight at 18 ˚C with shaking at 180 rpm.

### Protein purification and sample preparation

For the purification of the MD of RuV, the cells were harvested by centrifugation that was performed at 4 ˚C and at 8,000 rpm for 10 min (Thermo Scientific^®^, Sorvall Lynx 6000). Then, the cell pellet was resuspended with 25 mL lysis buffer (10 mM Imidazole, 50 mM Tris pH 8, 500 mM NaCl) and 10 µL of protease inhibitor cocktail (Sigma Aldrich^®^ P8849), 10% glycerol and 2 mM DTT. After the resuspended cells were sonicated (PMisonix^®^, Sonicator 4000), 50 µL DNase (1.6 mg/mL) were added to the suspension, which was further incubated for 10 min on ice. The cell extract was centrifuged at 4 ˚C at 14,000 rpm for 30 min. The soluble fraction containing the His_6_-tagged RuV MD was filtrated with a 0.22 μm membrane filter and was loaded onto a 5 mL Histrap^TM^FF affinity column (Cytiva) that had been previously equilibrated with 0.1 M NiSO_4_·6H_2_O and 5 column volumes (CV) lysis buffer. The protein was eluted using a step gradient with increasing concentrations of imidazole (10, 20, 40, 100, 200, 400 mM imidazole in buffer containing also 50 mM Tris pH 8, 500 mM NaCl). RuV MD eluted mostly in 100 and 200 mM imidazole-containing buffers. Using an Amicon^®^ Ultra 15 mL Centrifugal Filter membrane (nominal molecular weight cutoff 10 kDa), the protein was concentrated to a final volume of 1 mL, and as wl buffer exchange was performed from the imidazole-containing buffer to NMR buffer containing 50 mM NaPi pH 7.6, 50 mM NaCl, 2 mM EDTA, and 2 mM DTT. The concentrated protein sample was then loaded to a Superdex^TM^ 75 10/300 GL (GE Healthcare) column, previously equilibrated with NMR buffer, to remove any impurities. The elution fractions were analyzed by SDS-PAGE (17%) and Coomassie staining, and the ones containing the pure protein were pooled together and concentrated to a final volume of 500 µL. The final NMR samples had a final volume of 552 µL and were prepared by adding 1 µL of protease inhibitor cocktail (Sigma Aldrich^®^ P8849), 10% D_2_O and 0.25 mM DSS (4,4-dimethyl-4-silapentane-1-sulfonic acid - Sigma Aldrich^®^) used as an internal ^1^H chemical shift standard. Apart from the RuV MD apo state, the ADPr bound state in a molar ratio of RuV MD:ADPr − 1:5 (Sigma Aldrich^®^) was studied. The concentration of the double-labeled ^15^N, ^13^C RuV MD samples was 0.82 mM for the free form and 0.62 mM for the ADPr bound form while the ^15^N RuV MD samples were at 0.6 mM for the free form and at 0.45 mM for the ADPr bound form.

### NMR data acquisition and processing

All NMR experiments were recorded at 298 K on a Bruker Avance III High-Definition four-channel 700 MHz NMR spectrometer equipped with a cryogenically cooled 5 mm ^1^H/^13^C/^15^N/D Z-gradient probe (TCI). Table 1 provides an overview of the NMR experiments and corresponding main parameters acquired for backbone and side-chain assignments for both free and ADPr bound forms. The assignments of RuV MD in both states were obtained through analyzing the subsequent set of heteronuclear experiments: 2D ^1^H,^15^N HSQC and 2D ^1^H,^13^C HSQC, 3D HN(CO)CA, 3D HNCA, 3D HN(CO)CACB, 3D HNCACB, 3D HN(CA)CO, 3D HNCO, 3D HNHA, 3D HBHA(CBCACO)NH, 3D aliphatic (H)CCH–TOCSY and 3D ^1^H,^15^N NOESY, 3D ^1^H,^13^C aliphatic NOESY and 3D ^1^H,^13^C aromatic NOESY. All NMR data were processed using TopSpin 3.7.0 (Bruker Biospin) and analyzed using CARA 1.9.1.7 (Keller 2004).

**Table 1:** List of NMR experiments acquired at 700 MHz spectrometer at 298 K and corresponding main parameters used for backbone and side-chain assignments for RuV apo (**a**) and ADPr bound (**b**) form.

	Time domain data size (points)			Spectral width/Carrier frequency (ppm)			NS	Delay time (s)
	**t1**	**t2**	**t3**	**F1**	**F2**	**F3**		
(a) RuV apo								
^1^H,^15^N HSQC	256	2048		44/120 (^15^N)	14/4.7 (^1^H)		4	1
^1^H,^13^C HSQC	512	2048		160/80 (^13^C)	14/4.7 (^1^H)		32	1
CBCANH	96	40	1024	72/39 (^13^C)	44/117 (^15^N)	14/4.7 (^1^H)	32	1
CBCA(CO)NH	96	40	1024	72/39 (^13^C)	44/117 (^15^N)	14/4.7 (^1^H)	32	1
HNCA	80	40	1024	42/55 (^13^C)	44/117 (^15^N)	14/4.7 (^1^H)	8	1
HN(CO)CA	80	40	1024	42/55 (^13^C)	44/117 (^15^N)	14/4.7 (^1^H)	16	1
HNCO	64	40	1024	18/175 (^13^C)	44/117 (^15^N)	14/4.7 (^1^H)	8	1
HN(CA)CO	64	40	1024	18/175 (^13^C)	44/117 (^15^N)	14/4.7 (^1^H)	8	1
HNHA	48	96	1024	35/117 (^15^N)	14/4.7 (^1^H)	14/4.7 (^1^H)	16	1
HBHA(CBCACO)NH	112	40	1024	8/4.7 (^1^H)	44/117 (^15^N)	14/4.7 (^1^H)	16	1
(H)CCH-TOCSY	128	48	1024	80/39 (^13^C)	80/39 (^13^C)	14/4.7 (^1^H)	16	1
^1^H, ^15^N NOESY	232	48	2048	14/4.7 (^1^H)	40/117 (^15^N)	14/4.7 (^1^H)	16	1
^1^H, ^13^C NOESY aliphatic	192	64	1024	14/4.7 (^1^H)	80/39 (^13^C)	14/4.7 (^1^H)	8	1
^1^H, ^13^C NOESY aromatic	144	32	2048	14/4.7 (^1^H)	39/127 (^13^C)	14/4.7 (^1^H)	8	1
(b) RuV ADPr bound								
^1^H,^15^N HSQC	256	2048		44/120 (^15^N)	14/4.7 (^1^H)		4	1
^1^H,^13^C HSQC	512	2048		160/80 (^13^C)	14/4.7 (^1^H)		32	1
CBCANH	96	40	1024	72/39 (^13^C)	44/117 (^15^N)	14/4.7 (^1^H)	32	1
CBCA(CO)NH	96	40	1024	72/39 (^13^C)	44/117 (^15^N)	14/4.7 (^1^H)	32	1
HNCA	80	40	1024	42/55 (^13^C)	44/117 (^15^N)	14/4.7 (^1^H)	8	1
HN(CO)CA	80	40	1024	42/55 (^13^C)	44/117 (^15^N)	14/4.7 (^1^H)	16	1
HNCO	64	40	1024	18/175 (^13^C)	44/117 (^15^N)	14/4.7 (^1^H)	8	1
HN(CA)CO	64	40	1024	18/175 (^13^C)	44/117 (^15^N)	14/4.7 (^1^H)	8	1
HNHA	48	96	1024	44/120 (^15^N)	14/4.7 (^1^H)	14/4.7 (^1^H)	16	1
HBHA(CBCACO)NH	112	40	1024	8/4.7 (^1^H)	44/117 (^15^N)	14/4.7 (^1^H)	16	1
(H)CCH-TOCSY	128	48	1024	80/39 (^13^C)	80/39 (^13^C)	14/4.7 (^1^H)	16	1
^1^H, ^15^N NOESY	232	48	2048	14/4.7 (^1^H)	44/120 (^15^N)	14/4.7 (^1^H)	8	1
^1^H, ^13^C NOESY aliphatic	192	64	1024	14/4.7 (^1^H)	80/39 (^13^C)	14/4.7 (^1^H)	8	1
^1^H, ^13^C NOESY aromatic	144	32	2048	14/4.7 (^1^H)	39/127 (^13^C)	14/4.7 (^1^H)	8	1

### Extent of assignments and data deposition

**Fig. 2:**
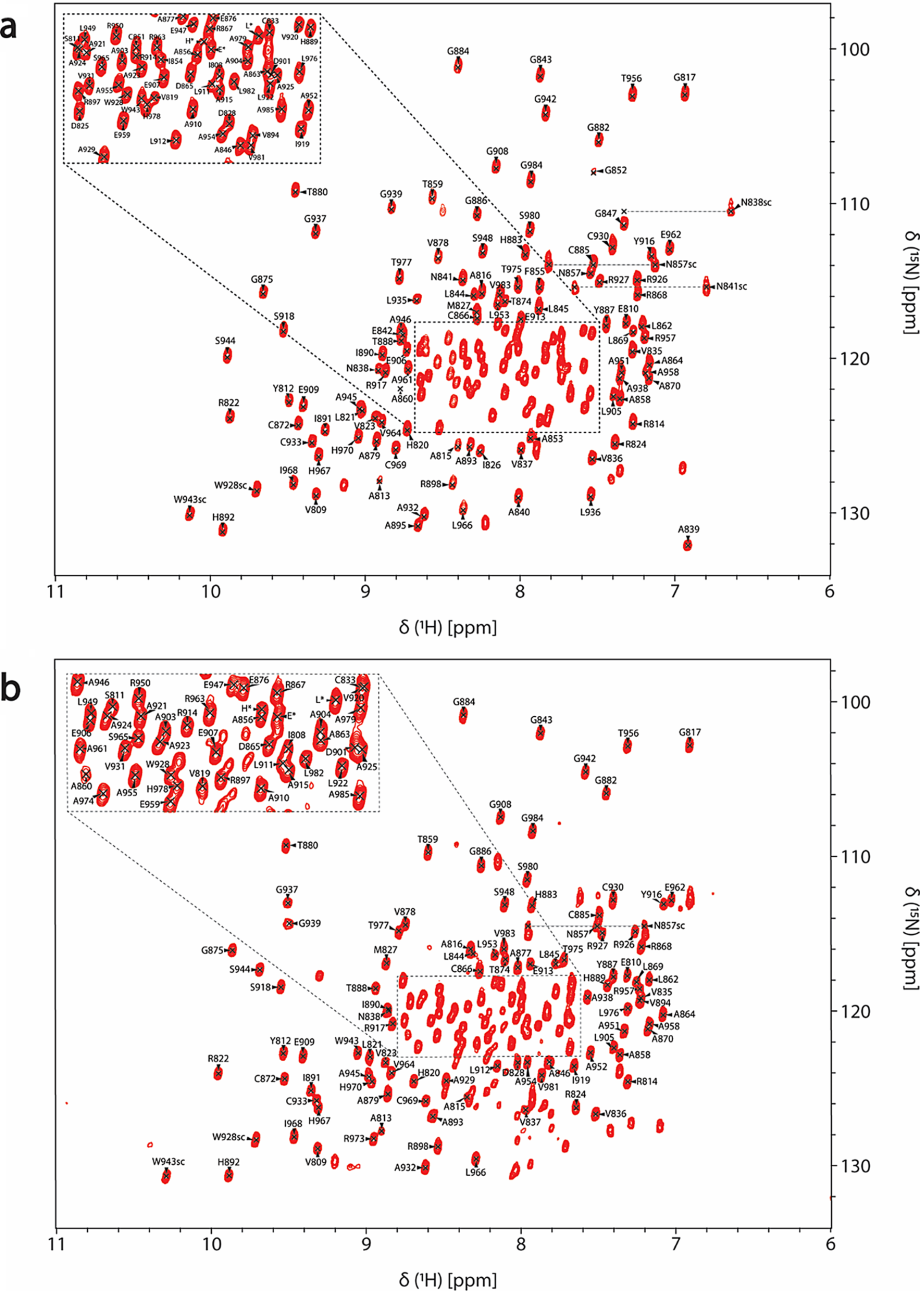
2D ^1^H,^15^N HSQC assigned spectrum of the (**a**) 0.82 mM ^15^N,^13^C RuV MD in the free state and (**b**) 0.62 mM ^15^N,^13^C RuV MD in the presence of ADPr (molar ratio RuV MD:ADPr − 1:5) obtained at 298 K. The reported amino acids for RuV MD are numbered in accordance with the native sequence of the multi-domain p150 protein. The resonances corresponding to Asn and Trp side-chains are labeled with “sc”. The assigned residues arising from cloning artifacts are labeled with asterisks. The unassigned peaks of the spectra correspond to unassigned residues.

**Fig. 3:**
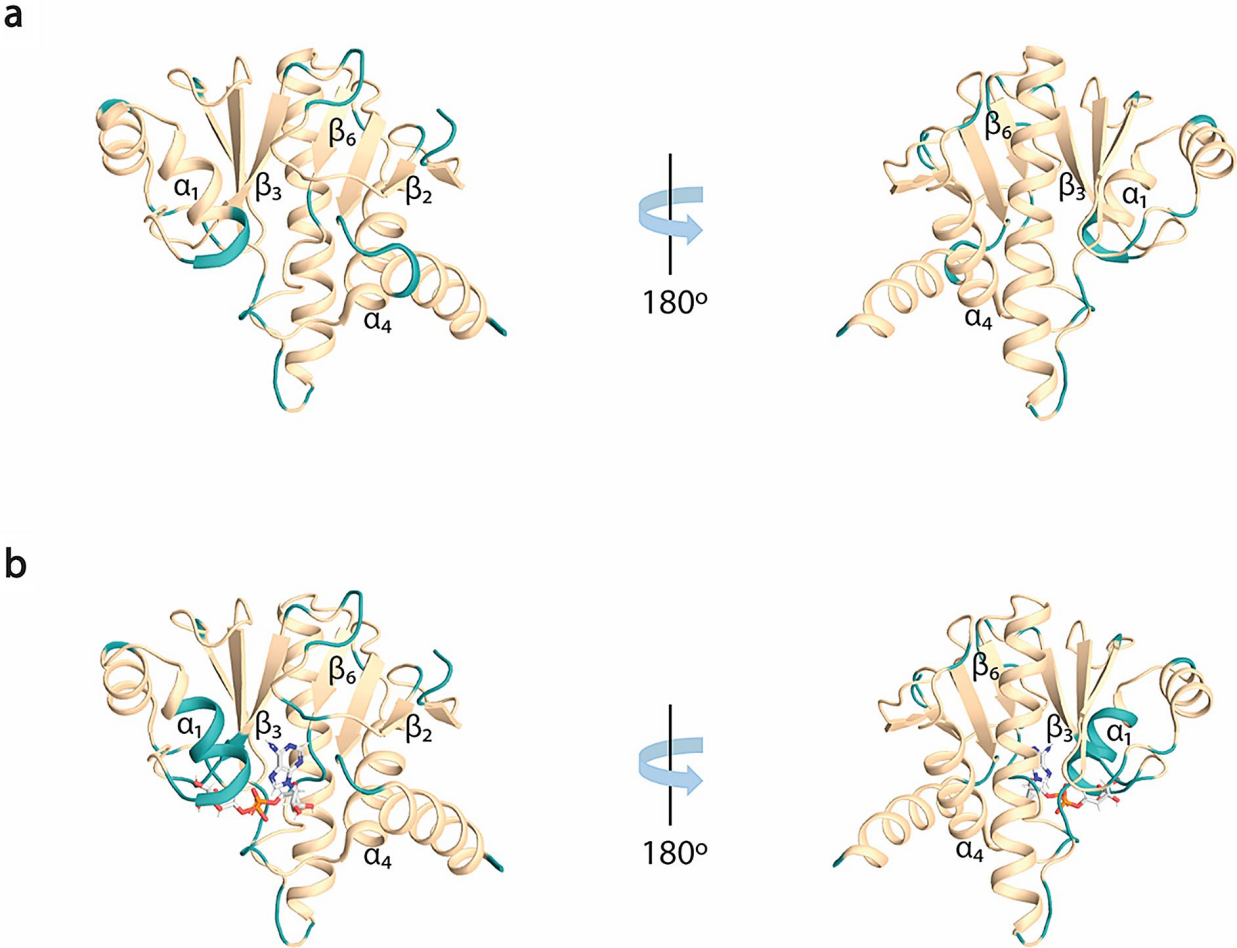
Illustration of the unassigned residues in ^1^H,^15^N HSQC spectrum for the two RuV MD forms. (**a**) Cartoon representation of RuV MD structure in the free state (PDB ID: 8P0C) and (**b**) ADPr bound (PDB ID: 8P0E) (Stoll et al. 2024) with missing residues colored in turquoise.

In both free (Fig. 2a) and ADPr bound (Fig. 2b) forms, the amide signals in the 2D ^1^H,^15^N HSQC spectra are well-dispersed, which is a sign of a well-folded protein in the absence and presence of ADPr. For the RuV MD apo, 92% of ^1^H^N^/^15^N backbone pairs, 93% of ^13^CO, 94% of ^13^Cα and 94% of ^13^Cβ chemical shifts as well 69% of the total atoms of side chains of native protein sequence (806-985 a.a.), were assigned. Regarding the ADPr bound form, 84% of the ^1^H^N^/^15^N backbone pairs, 87% of ^13^CO, 88% of ^13^Cα and 88% of ^13^Cβ chemical shifts as well 65% of the total atoms of side chains of native protein sequence were assigned. The unassigned residues in 2D ^1^H,^15^N HSQC spectrum of RuV MD in the apo form, except for the 13 prolines present in the sequence, correspond to the residues S806 to D807, G832, K834, S848 to V850, A861, R900, V940, Y941, D972 to A974 and the His_6_-tag. In the ADPr bound form, apart from the unassigned residues in apo form with the exception of R973-A974, the residues D825-I826, A839 to E842, G847 to F855, A895 and L935-L936 were also not assigned. These residues are in the loops of RuV MD which are mainly forming the binding site of ADPr. Specifically, the residues D825-I826 located in the β2-β3 loop, that are conserved in most viral macro domains and are responsible for the stabilization of the adenine group of ADPr, disappeared in the presence of ADPr (Fig. 3b). Moreover, the residues at the end of β3 and the catalytic loop β3-α1, A839 to E842, as well as the residues G847 to F855 located in α1, which interact with the distal ribose of ADPr, are broadened beyond detection upon ADPr binding (Fig. 3b). Furthermore, the residues V940-Y941 located in the β6-α4 loop that bind the phosphate groups of ADPr were not assigned in both apo and bound states while the residues L935-L936 located in the same loop disappeared with the ADPr addition (Fig. 3b). The appearance and disappearance of the signals in the ADPr bound form suggest conformational exchange in these regions to accommodate the ligand. Similar observations were made in the NMR studies of a human MD belonging to hPARP14 and of the coronaviruses’ MDs from SARS-CoV and SARS-CoV-2 (Cantini et al. 2020; Fourkiotis et al. 2022; Tsika et al. 2022). In contrast, for the MD of alphavirus MAYV, the opposite effect took place; the structure upon ADPr binding became more rigid, and these conserved residues were observable, which was not true for the apo state (Tsika et al. 2019).

**Fig. 4:**
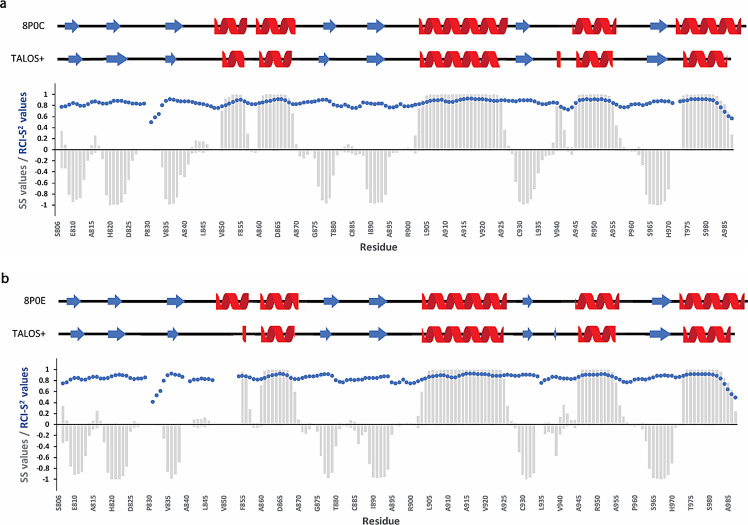
Secondary structure and flexibility analysis of RuV MD in (**a**) apo and (**b**) ADPr bound state. Comparison of secondary structure analysis from the crystal structures (upper; 8P0C and 8P0E, respectively) with secondary structure prediction based on NMR experimental data using the TALOS+ server (Shen et al. 2009) (middle) shows good agreement for both RuV MD forms. Red cartoon indicates *α*-helix and blue arrow *β*-strand. In the plot (lower), the *α*-helix and *β*-strand SS values predicted using the TALOS+ server for each residue are shown as positive and negative values, respectively. The negative SS values for *β*-strands propensities are given for illustration purposes. Values close to 1 or -1 indicate a high possibility for *α*-helix and *β*-strand, respectively. The flexibility of RuV MD is also depicted as Random Coil Index (RCI) derived S^2^ (RCI-S^2^) values predicted by the TALOS+ server (Shen et al. 2009) and shown with blue dots. The reported amino acids for RuV MD are numbered in accordance with the native sequence of the multi-domain p150 protein.

The secondary structure prediction for both apo and ADPr bound forms was achieved based on the chemical shift assignments of the backbone atoms ^1^H^N^, ^15^N, ^13^CO, ^13^Cα, ^1^Hα and ^13^Cβ using the TALOS+ server (Shen et al. 2009). Both forms adopt an α/β/α three -layered sandwich fold, consisting of 7 beta strands and 5 alpha helices with β/β/β/α/α/β/β/α/β/α/β/α topology (Fig. 4), typical for the macro domain fold, with no significant differences between the two states. Moreover, the resulting secondary elements were compared with the existing crystal structures of the RuV MD (apo form PDB ID: 8P0C and bound form PDB ID: 8P0E) (Stoll et al. 2024) and they are in good agreement, as shown in Fig. 4. The flexibility of RuV MD in the apo and ADPr bound states was also predicted and described as Random Coil Index (RCI) derived S^2^ values (RCI-S^2^) (blue dots in Fig. 4). Most RCI-S^2^ values are close to 1, a sign of a rigid structure, except for the N- and C-termini and the loops in which the values are lower, a sign of more flexible regions.

All the chemical shift data of RuV MD in both free and ADPr bound forms have been deposited in the Biological Magnetic Resonance Bank (https://bmrb.io) under accession numbers 52869 and 52870, respectively.

## Data Availability

All the chemical shift values of RuV MD apo and ADPr bound forms were deposited in the Biological Magnetic Resonance Data Bank (BMRB) under accession numbers 52869 and 52870, respectively.
